# The Clinical Impact of Recent Methamphetamine Exposure in Aneurysmal Subarachnoid Patients

**DOI:** 10.21203/rs.3.rs-2694424/v1

**Published:** 2023-03-31

**Authors:** Jeffrey R Vitt, Roger C Cheng, Jason Chung, Michael Travis Canton, Bo Zhou, Nerissa Ko, Karl Meisel, Ediberto Amorim

**Affiliations:** UC Davis: University of California Davis; Rutgers Robert Wood Johnson Medical School; University of California San Francisco Department of Neurological Surgery; University of California San Francisco; University of California San Francisco Weill Institute for Neurosciences; University of California San Francisco Weill Institute for Neurosciences; University of California San Francisco Weill Institute for Neurosciences; University of California San Francisco Weill Institute for Neurosciences

**Keywords:** aneurysmal subarachnoid hemorrhage, vasospasm, delayed cerebral ischemia, methamphetamine, transcranial doppler, digital subtraction angiography, subarachnoid hemorrhage, aneurysmal, vasospasm, intracranial, ultrasonography, doppler, transcranial, methamphetamine, cerebral angiography

## Abstract

**Background:**

Methamphetamines (MA) are a frequently used drug class with potent sympathomimetic properties that can affect cerebral vasculature. Conflicting reports in literature exist about the effect of exposure to MA on vasospasm risk and clinical outcomes in aneurysmal subarachnoid hemorrhage (aSAH). This study aimed to characterize the impact of recent MA use on the timing, severity and features of vasospasm in aneurysmal subarachnoid as well as neurological outcomes.

**Methods:**

We retrospectively screened 441 consecutive patients admitted to a tertiary care hospital with a diagnosis of SAH who underwent at least one cerebral digital subtraction angiogram (DSA). Patients were excluded if no urinary toxicology screen was performed within 24 hours of admission, if there was a diagnosis of non-aneurysmal SAH, or if ictus was greater than 72 hours from hospital admission. Vasospasm characteristics were collected from DSA and transcranial doppler (TCD) studies and demographic as well as clinical outcome data was abstracted from the chart.

**Results:**

129 patients were included and 24 tested positive for MA. Among the 312 excluded patients, 281 did not have a urinary toxicology screen and 31 had a non-aneurysmal pattern of SAH or ictus occurring greater than 72 hours from hospital admission. No significant differences were found in respect to patient age, sex, or admission Hunt and Hess Score or Modified Fisher Scale based on MA use. There was no difference in the severity of vasospasm or time to peak severity using either TCD or DSA criteria on multivariate analysis. Aneurysms were more likely to be in the anterior circulation for both groups, however the MA cohort experienced less vasospasm involving the anterior circulation and more isolated posterior circulation vasospasm. There was no difference in delayed cerebral ischemia (DCI) incidence, length of ICU stay, need for ventriculoperitoneal shunt placement, functional outcome at discharge or hospital mortality.

**Interpretation:**

Recent MA use was not associated with worse vasospasm severity, time to vasospasm, or DCI in aSAH patients. Further investigations about localized MA effects in the posterior circulation and impact on long-term functional outcomes are warranted.

## Introduction

Aneurysmal subarachnoid hemorrhage (aSAH) accounts for approximately 5% of strokes with an annual incidence of approximately 6–10 per 100,000 persons (1, 2). While the overall incidence of SAH has been decreasing globally over the past several decades, aSAH carries a high burden of mortality with a case fatality rate of 27–44% (2–5). Nearly half of SAH survivors will not achieve functional independence at one year and many continue to experience significant long-term deficits in memory, executive function, and language (3, 6).

Certain psychoactive substances such as methamphetamines (MA), tobacco, cocaine, and cannabis have been tied to the development of vasospasm and heightened risk of delayed cerebral ischemia (DCI) after SAH (7–10). Approximately 30% of SAH patients develop DCI, a diagnosis strongly associated with long-term neurologic disability and mortality (11–13). The underlying pathophysiology of DCI is likely multifactorial, and includes microcirculatory disfunction, loss of autoregulation, cortical spreading depolarizations, microthrombi formation leading to cerebral hypoperfusion, metabolic mismatch, and cerebral infarction (14). Large vessel vasospasm is a common finding in SAH patients and is also a potential contributor to DCI by impairing cerebral blood flow (CBF) and vascular adaptation to metabolic demand (12, 15). Psychoactive drugs can have direct vascular effects in cerebral arteries, but the pathways activated by these drugs contributing to DCI after SAH have not been elucidated. (7, 16, 17).

Methamphetamines are synthetic amphetamine-type stimulants with potent sympathomimetic properties (18, 19). Its use has been associated with a variety of cerebrovascular complications, including increased risk for aneurysm formation and rupture (17, 20, 21). Contradicting reports exist however on the impact of MA use on the development of vasospasm and DCI in aSAH patients, however these studies did not delineate differences between chronic and recent MA use (8, 22, 23). In this study, we aimed to characterize the influence of recent MA use on the time course and severity of vasospasm as well as DCI occurrence following SAH leveraging multimodal imaging.

## Methods

### Patient Population

Electronic health records (EHR) were reviewed between December 28, 2011 and January 1, 2019 and for all subjects admitted to the University of California San Francisco (UCSF) Medical Center with a diagnosis non-traumatic SAH based on ICD codes (430 and 160.9) and at least one digital subtraction angiography performed. Subjects were excluded if they were younger than 18 years of age, did not have an aneurysmal source for SAH, did not have a urine toxicology screen within 24 hours of admission, and if time of aneurysmal rupture was greater than 72 hours from admission.

### Clinical Data

Demographics including age, sex, admission status (direct from emergency department versus transfer from outside facility), onset of SAH, aneurysm location and urine toxicology results were collected from EHR. If subjects tested positive for amphetamines on urine toxicology screening on admission, they were included in the MA cohort. Hunt and Hess (HH) Classification was abstracted from the admission documentation and the Modified Fisher Scale (mFS) was determined from admission head computed tomography (CT). Information on external ventricular drain (EVD) placement, duration of drainage as well as if a ventriculoperitoneal shunt (VPS) placement was abstracted. Hospital and intensive care unit (ICU) length of stay as well as discharge disposition (home, acute rehabilitation, skilled nursing facility, acute care facility, hospital transfer or death) and Modified Rankin Scale (mRS) at time of discharge were recorded. A good functional outcome was considered as a mRS ranging 0–3 (i.e., no symptoms to at least able to walk unassisted).

Delayed cerebral ischemia was abstracted from the chart and defined as either a new focal neurologic impairment or decrease of at least 2 points on the Glasgow Coma Scale lasting for at least one hour and was not immediately apparent after aneurysm occlusion nor could be attributed to another cause (24). Delayed infarction on either CT or magnetic resonance imaging (MRI) not attributable to surgical procedures or endovascular treatment was also considered DCI.

Severity of vasospasm on DSA was scored by dual board-certified Neuroradiology and Neurointerventional Radiology faculty and rated as absent, mild, moderate or severe vasospasm (25). Subjects underwent at least one DSA may have had repeat angiograms as clinically indicated. Transcranial doppler (TCD) assessment is routinely pursued for vasospasm surveillance in our institution, with results interpreted by an attending Vascular Neurologist with certification in Neurosonology. For statistical analysis, vasospasm was considered anterior predominant or posterior predominant depending on where the most severe vasospasm was recorded on either DSA or TCD. In cases where the severity of vasospasm was equal in the anterior and posterior circulation the vasospasm was considered multifocal.

### Transcranial Doppler Testing

TCD was performed as clinically indicated by a Registered Vascular Technologist with specialization in TCD using a 2-MHz probe and ST3 Transcranial Doppler (Spencer Technologies, Redmond, WA, USA). Vasospasm was classified as mild, moderate and severe by mean flow velocities (MFV) of 120–139 cm/s, 140–179 cm/s, and > 180 cm/s with corresponding Lindegaard ratios of 3.0–3.99, 4.0–5.99, and > 6.0 in the anterior circulation vessels and basilar/vertebral artery ratios (MFV Basilar Artery)/(Right Vertebral Artery + Left Vertebral Artery)/2 of 2.7–3.29, 3.3–4.3, and > 4.3 in the posterior circulation. When disagreement between MFV and ratio measurements was identified, final determination was made according to whichever value corresponded to the lower overall severity.

## Statistical Analysis

Continuous variables and categorical variables are reported as medians with interquartile ranges and frequencies with percentages. We utilized the Shapiro-Wilk test to evaluate normality distribution. Chi-square was used to compare categorical data. The Student’s t-test was used for variables with normal distribution and Mann-Whitney for non-normally distributed data. Ordinal logistic regression analysis was used to compare the incidence of DCI as well as maximal vasospasm severity and time from SAH onset to peak vasospasm using both DSA and TCD based on MA use. The following known risk factors for vasospasm were assessed using univariable ordinal logistic regression: age, HH and mFS. Variables with a P of ≤ 0.1 in the univariable analysis were included in the multivariable ordinal logistic regression model. A multivariable logistic regression was pursued for occurrence of DCI. We repeated the ordinal logistic regression models only including patients who developed severe vasospasm by DSA or TCD. Data were analyzed using R version 3.5.1 (R Foundation for Statistical Computing, Vienna, Austria). Ordinal regression was performed using the ordinal package.

## Results

**Figure 1:**
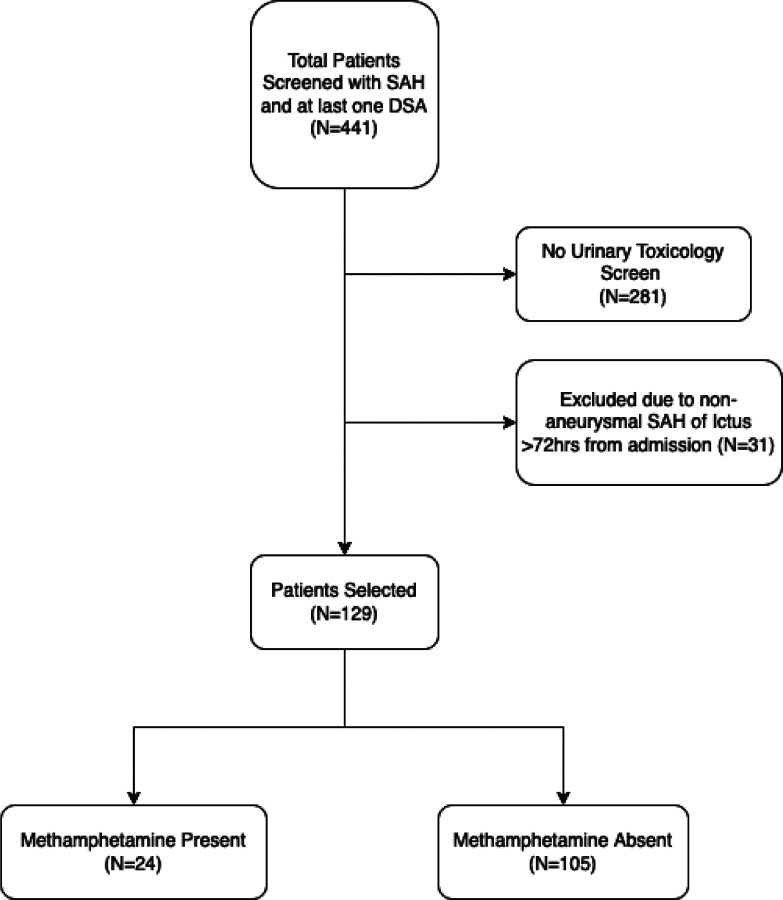
Flow chart of patient selection. SAH: subarachnoid hemorrhage, DSA: digital subtraction angiography.

A total of 441 subjects with a primary diagnosis of non-traumatic SAH and underwent at least one DSA were screened. Of these, 281 subjects were excluded due to lack of urinary toxicology screen performed within 24 hours of admission. An additional 31 subjects were excluded due to non-aneurysmal source of hemorrhage or ictus greater than 72 hours from admission. The remaining 129 subjects were included for primary analysis, including 24 subjects in the MA present group (Fig. 1). There was no significant difference in MA present and MA absent groups regarding age (50.1 vs. 51.4, p = 0.62), female sex (66.7% vs. 63.8%, p = 0.87), admission HH (median 3 for both groups), or mFS (median 4 for both groups). Most subjects were transferred from an outside facility (95.8% vs. 87.6%, p = 0.42). Overall, subjects with MA use had more posterior circulation aneurysms (29.2% vs. 20%, p = 0.19) and EVD insertion (75% vs. 61.9%, p = 0.33) though neither factor reached statistical significance (Table 1).

**Figure 2:**
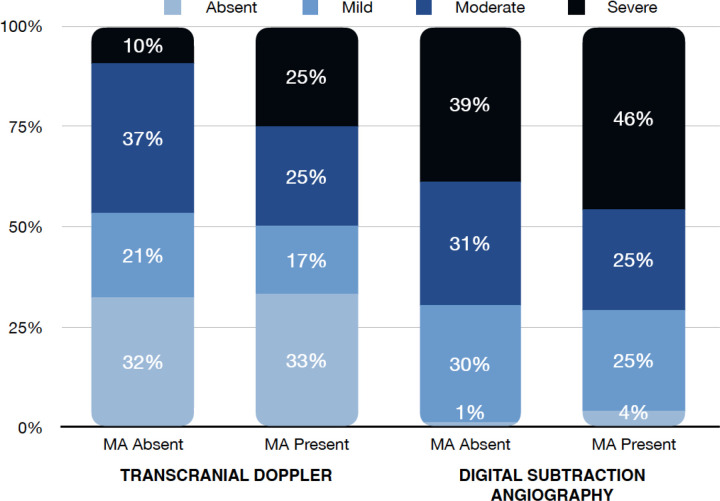
Severity grade of vasospasm by transcranial doppler and digital subtraction angiography divided into methamphetamine (MA) absent or present cohorts.

While vasospasm of any grade was more commonly diagnosed with DSA than TCD, there was no difference in vasospasm incidence for the MA present or MA absent groups for either modality (95.8% vs 99%, p = 0.82) and (66.7% vs. 67.6%, p = 1), respectively. When evaluating only cases of severe vasospasm, similarly there was no difference in respect to MA use with DSA (45.8% vs 39%, p = 0.7) or TCD (25% vs. 9.5%, p = 0.08) (Fig. 2). Time to peak vasospasm was similar in both groups on DSA (6.5 vs 7 days, p = 0.32) or TCD (5.5 vs. 6 days, p = 0.95). There was also no notable difference in the incidence of DCI between the two groups (20.8% vs. 21.9%, p = 1). Both groups underwent a similar amount of DSA procedures (median 3.5 vs. 3, p = 0.37) throughout the hospital stay and each group had a median of 5 TCD examinations performed. In univariate analysis, vasospasm of any severity involving the anterior circulation was more commonly observed in the MA absent group (79.2% vs. 94.3%, p = 0.03) and there was a higher proportion of isolated posterior circulation vasospasm in the MA present group (16.7% vs 4.8%, p = 0.06) though this did not reach statistical significance.

Duration of EVD use was similar in both groups (17 vs. 18 days, p = 0.63) as well as the rate of VPS placement (25% vs. 21%, p = 0.93), length of ICU stay (19.5 vs. 18 days, p = 0.3) and hospital length of stay (23.5 vs. 20 days, p = 0.06). There was no difference in rates of discharge to home or acute rehab (45.8% vs. 60%, p = 0.3), favorable functional outcomes at discharge (41.7% vs. 55.2%, p = 0.31) or hospital mortality between both groups (4.2% vs 6.7%, p = 1).

Univariate analysis revealed increased risk of severe vasospasm diagnosed by TCD in the MA present group, but that was not observed with DSA. In multivariable regression analysis, MA use was not associated with the development of severe vasospasm using either TCD or DSA nor incidence of DCI. In contrast, younger age as well as higher HH and mFS on admission were associated with increased risk of severe vasospasm development diagnosed by DSA, though not TCD, in univariate and multivariable analysis. With respect to DCI, univariate analysis revealed an association with higher HH, mFS, and severe vasospasm diagnosed with TCD or DSA. On multivariable analysis, only admission HH and severe vasospasm diagnosed on DSA were found to be associated with DCI occurrence (Table 4).

## Discussion

In this retrospective analysis of subjects admitted with aSAH, we found that recent exposure to MA was not associated with the development, time course or severity of cerebral vasospasm nor with the incidence of DCI. Contrary to previously published reports, our study exclusively evaluated subjects presenting with MA present on urine toxicology screening, therefore it was better suited to assess the potential direct interaction of systemic MA and associated metabolites with the development of vasospasm and DCI following SAH (8, 22, 23). Using a multimodal approach with TCD and DSA, we were able to thoroughly characterize the incidence, distribution and chronicity of vasospasm, finding less frequent development of anterior circulation vasospasm and a trend towards more isolated posterior vasospasm in subjects with recent MA use. While there was a trend towards worse functional outcome and less discharge to home or acute rehabilitation in the MA present group, these results were not statistically significant and there was no overall impact on hospital length of stay, VPS placement, or hospital mortality. These findings indicate that active use of MA may not be associated with increased risk of vasospasm and DCI in SAH, and that care for these patients should follow standard protocols for surveillance.

Patient’s age at the time of SAH presentation was similar between subjects with or without recent MA exposure in our study. The median age at presentation was 55, ranging from 28 to 64 years. This is in contrast to prior published cohorts of patients using MA who were admitted at a much younger age on presentation, with a median age generally in the fourth decade of life (8, 22, 26). As younger age is a risk factor for vasospasm, it is possible that age might have confounded this association in previous reports. Our findings may suggest that MA use is more common in older populations compared to historical cohorts or reflect local demographics in the Northern California region. Internationally, MA use has risen sharply across different segments of the population with production growing worldwide (18). In the United States, MA use has increased sharply over the past decade, particularly among individuals using other substances of abuse, and is responsible for nearly a quarter of drug-related treatment admissions (19, 27). MA effects the central nervous system by increasing the release of serotonin, norepinephrine and dopamine into the synaptic cleft as well as inhibiting neurotransmitter degradation thereby increasing postsynaptic activity (17). Chronic MA use has wide ranging deleterious systemic effects, particularly involving the cardiovascular and cerebrovascular systems related to catecholamine toxicity, vessel inflammation, vascular remodeling and accelerated atherosclerosis (28, 29).

Given conflicting prior reports on the impact of MA use and cerebral vasospasm following SAH, we sought to better characterize the incidence, time course and severity of vasospasm using repeated surveillance monitoring with TCD and DSA (8, 22, 23). It is well known that MA exerts vasoconstriction properties on the cardiopulmonary vasculature and has been linked with increased mortality and morbidity (29). In animal studies, exposure to IV MA induces a potent and prolonged cerebral vasospasm response involving both the large and small vessels leading to sustained diminished CBF and ultimate infarction (16, 30). Vasospasm related to aSAH is a well-described clinical phenomena, occurring in around 70% of patients by day 7 post-bleed, and when severe (> 50% narrowing of luminal diameter) is an established risk factor for the development of DCI, cerebral infarction and worse neurologic outcomes (7, 15, 31, 32). Molecular studies have illuminated differential expression of nitric oxide, enthothelin-1, renin-angiotensin system as well as calcium and thrombin signaling in patients with SAH associated vasospasm suggesting a multitude of diverse pathways are responsible for the clinical phenotype (33). Despite the known vasoconstrictive properties of MA, the impact of acute or recent MA exposure on the development and characteristics of vasospasm related to aSAH has not been well studied. Prior published studies on MA use and SAH found no overall difference in the incidence of DCI or vasospasm after adjusting for age, however these studies included subjects who had either a reported history of MA use or positive toxicology results (8, 22). In contrast, our study only evaluated subjects with MA present on urine toxicology screen at hospital admission, thus indicating a recent exposure. This stricter approach was pursued to determine the impact of systemic MA and metabolites on the development of vasospasm and DCI following SAH. Our results confirm that recent MA exposure does not influence the incidence, time course or severity of SAH associated vasospasm. With multivariable analysis we found that young age and higher mFS and HH on admission predicted the development of severe vasospasm on DSA in keeping with prior published reports (7, 34). Likewise, we did not find any association between MA use and DCI, however severe vasospasm on DSA and higher HH on admission were associated with increased incidence in multivariable analysis as expected. Severe vasospasm on TCD was predictive of DCI in univariate though not in multivariable analysis.

This study also evaluated location of vasospasm in MA use, a feature omitted in previous reports (8, 20, 22). While anterior circulation vasospasm was the most commonly observed location for either cohort, there was a significant difference in regional vasospasm distribution such that the MA present group had a lower incidence of anterior circulation involvement with more isolated posterior circulation vasospasm, an association that has not previously been reported (8, 20, 22). The basilar and vertebral arteries are known to exhibit a higher propensity towards disrupted cerebral autoregulation and alterations in vascular tone due to a more heterogenous distribution of sympathetic innervation compared to the anterior circulation (35). This increased susceptibility is thought to underlie why posterior reversible encephalopathy syndrome (PRES) often manifests with posterior circulation predominant vasoconstriction and can be triggered by various factors including vasoactive substances, uncontrolled hypertension, immune modulating agents as well as renal disease (36–38). It is possible that shared pathophysiology between alterations in vascular tone from SAH and PRES may confer increased predilection of vasospasm in the posterior circulation in the presence of a vasoactive substance such as MA, but given our small sample this association requires confirmation on studies involving a larger patient population. The observed decrease in anterior circulation vasospasm and higher incidence of isolated posterior vessel vasospasm with MA could also be explained by the slightly higher proportion of posterior circulation aneurysms in this group. Of the seven (29.2%) patients in the MA present group who had a ruptured posterior circulation aneurysm, three (42.9%) developed vasospasm predominantly involving the posterior circulation. In contrast, posterior circulation aneurysms were noted in 21 (20%) of patients in the MA absent cohort, with three (14.3%) having vasospasm exclusively involving the posterior circulation and 12 (57.1%) with both posterior and anterior circulation vasospasm. Use of MA has been linked with increased risk of aneurysm formation as well as accelerated growth, abnormal morphological changes and rupture, likely owing to direct toxic effects on the vasculature leading to inflammation and impaired vessel wall integrity (17, 39, 40). Although multiple prior studies did not find an interaction between MA use and aneurysm location, a recently published large case series of MA related aneurysms found that while anterior circulation aneurysms were more common overall (77%), posterior aneurysms were disproportionately more likely to present with rupture compared to anterior aneurysms (65% vs 32%) and rupture despite smaller size (8, 20–22). These findings are consistent with our cohort results, which showed an increased prevalence of posterior aneurysms in MA present group presenting with aneurysm rupture.

In our study, we found no difference in functional outcomes, discharge disposition to home or acute rehabilitation, mortality or hospital and ICU lengths of stay with MA use. Prior studies involving the impact of MA use in ICH subjects found an increased length of hospital stay, largely related to need for anti-hypertensive medication infusions, though not mortality or functional independence (26, 41). In contrast, Beadell and colleagues reported a trend towards worse functional outcomes in MA users presenting with SAH which reached statistical significance after age matched control sub-analysis (8). Similarly, using a large SAH trial registry, Moon an colleagues found that after one and three years follow up, MA users had no increased mortality though had higher rates of severe disability and functional dependence compared to controls, despite no difference in rates of clinical or radiographic vasospasm (22). These findings suggest that, for mechanisms which are currently unknown, MA use may exert lasting impacts that hinder neurorecovery following SAH.

There are several limitations to our study. First, it is a single-center retrospective analysis, limiting the generalizability of our findings. For the MA present cohort, we included all subjects with a positive urine toxicology screen for amphetamines on admission, and therefore it is possible some patients may have been misclassified due to the exposure to alternate prescription or recreational substances (42). Furthermore, since we only included toxicology positive subjects in the MA present group, the influence of more remote and chronic MA use could not be determined accurately through retrospective review. We also did not take into consideration other substances, with or without concomitant use with MA, which may have also impacted vascular characteristics and functional outcomes (19). Moreover, subjects were selected only if they had at least one DSA performed, what may have introduced a selection bias towards patients who were more likely to receive aggressive treatment. The use and frequency of TCD and DSA was not standardized and was left up to the clinical judgement of the treating team. Therefore, it is possible that vasospasm severity or high-risk clinical features influenced the frequency of the studies performed as well as the accuracy and characterization of vasospasm time-course and interventions. Our designation of vasospasm severity was made by both TCD and DSA modalities, which are known to have modest interrater agreement, particularly with non-severe vasospasm and in vessels outside of the MCA (43, 44).

## Conclusion

In patients presenting with aSAH, recent MA use does not impact the incidence, chronicity or severity of vasospasm nor does it influence the occurrence of DCI. Our study confirms prior established risk factors for vasospasm including young age, increased volume of hemorrhage as well as more severe clinical grade on presentation independent of MA exposure. These findings support the use of standard vasospasm surveillance practices in patients with recent MA exposure presenting with aSAH. The influence of recent MA use and distribution of vasospasm with possible predilection for the posterior circulation warrants further investigation in subsequent studies.
